# Challenges and Strategies in Managing Recurrent *Clostridioides difficile* Infection in Older Adults

**DOI:** 10.3390/geriatrics10060158

**Published:** 2025-12-02

**Authors:** Imaan Hirji, Divya John, Jeena Jith, Hiro Khoshnaw, Myooran Ganeshananthan

**Affiliations:** Royal Surrey NHS Foundation Trust, Guildford GU2 7XX, UK

**Keywords:** *Clostridioides difficile*, *C. diff* toxin, *C. diff* recurrence, faecal microbiome transplant, older adults, geriatrics, infection control, multimorbidity, antibiotics, infectious diseases

## Abstract

Background: *Clostridioides difficile* infections (CDIs) are caused by a Gram-positive, spore-forming bacillus and are defined by more than three episodes of watery diarrhoea per day. CDI is a major cause of morbidity and mortality in older adults, particularly over 65 years. Recurrent CDI leads to higher mortality and prolonged, debilitating illness. Case Presentations: This article presents two patients, aged over 80 years old, who developed recurrent CDI causing complicated and prolonged treatment courses. Patient 1 required an extended course of antibiotics for treatment of discitis and a congruent psoas abscess. Patient 2 developed CDI after multiple short courses of antibiotics for urinary tract infections (UTIs) in the context of multiple comorbidities. Both patients experienced three distinct episodes of CDI and were treated in collaboration with microbiology specialists. Following the third episode, both were successfully treated with oral capsule faecal microbiome transplants (FMTs). Their cases highlight the challenge of balancing systemic antibiotic use against CDI risk. Discussions: These cases underscore known risk factors for recurrent CDI, including advanced age and prolonged antibiotic exposure. Recurrence rates in patients over 65 can reach 58%. The British Society of Gastroenterology and Healthcare Infection Society support the use of FMTs in recurrent cases. Environmental decontamination, including terminal cleaning with sporicidal agents, is critical in reducing reinfection in hospital settings. Conclusions: Recurrent CDI in elderly patients reflects a complex interplay between infection control and managing comorbidities. New guidelines suggest that FMTs can significantly reduce morbidity and mortality. These cases emphasise the need for individualised, multidisciplinary care, adherence to guidelines, and further research to improve safe, effective CDI management in older adults.

## 1. Introduction

*Clostridioides difficile* (*C. diff*) is a Gram-positive, spore-forming anaerobic bacillus. Toxigenic strains produce toxins A and B, the key virulence factors responsible for clinical manifestation in *C. difficile* infection (CDI) [1]. Advanced age (>65 years), female sex, recent antibiotic use, use of gastric acid suppressing agents, enteral feeding, and hospitalisation are well established risk factors [2,3]. Certain antibiotics, particularly cephalosporins, quinolones, and clindamycin are associated with higher risk of CDI [2,3]. Hospitalisation increases exposure to antibiotics and contaminated environments, while also involving a vulnerable elderly population [4].

*C. difficile* is a natural part of the gut microbiome that is usually kept in check by other commensal flora [5]. Disruption to this microbiome, typically by antibiotic exposure, allows for spore germination and production of toxins, which leads to symptoms and colitis [5]. CDI is primarily transmitted via the faeco-oral route through resilient spores that persist in the environment, making infection control crucial in healthcare settings [6]. Clinical manifestations vary from mild, self-limiting diarrhoea to severe life-threatening complications including pseudomembranous colitis, toxic megacolon, and bowel perforation [6].

The ageing population experiences higher morbidity and mortality associated with CDI [7]. A European multicentre case–control study identified cancer, malnutrition, cachexia, renal impairment, and higher Charlson comorbidity index scores as significant predictors of 90-day mortality following CDI [8]. Recurrence remains a major clinical concern, affecting approximately 20% of patients after initial antibiotic therapy [9,10]. Of this cohort, up to 88% of recurrence results from relapse of the same strain, and the rest relapse from reinfection of a new strain [9,10]. Up to 25% of patients experience recurrent *C. diff* within 30 days of completing treatment [11].

This paper presents two cases of recurrent CDI in elderly patients, emphasising the challenges and consideration in managing this condition within a vulnerable population.

## 2. Case Reports

### 2.1. Patient 1

Patient 1 is an 86-year-old female who presented to hospital on 15 August 2024 following a fall with a few days’ history of generalised body aches. On admission to the hospital, she received a full fall work-up and was found to have an *Escherichia coli* (*E. coli*) bacteraemia, treated with a seven-day course of intravenous (IV) co-amoxiclav. The infection was initially attributed to a urinary tract infection; however, two negative urinary cultures and a normal renal ultrasound prompted further investigation for another source.

Despite antibiotics, two further blood cultures grew *E. coli*. During this period, she developed some back pain as well as diarrhoea. Due to the recent antibiotic use, she was tested for *C. diff*, and the test was both PCR- and toxin-negative. The diarrhoea was thought to be secondary to antibiotic use, as the patient had a known amoxicillin sensitivity.

With ongoing diarrhoea and back pain, further imaging was performed. Computed tomography (CT) thorax abdomen and pelvis showed toxic megacolon, small bowel wall thickening, and transverse colon dilatation, while MRI spine revealed T12/L1 discitis and a left psoas abscess causing canal effacement. Neurosurgery advised conservative management, and she began a prolonged antibiotic course (co-trimoxazole, piperacillin-tazobactam, and amoxicillin).

Antibiotics unfortunately worsened her diarrhoea and on 18 September 2024, a repeat stool test was PCR- and toxin enzyme immunoassay (EIA)-positive for *C. diff*. She completed a 10-day course of oral vancomycin with good symptomatic improvement. Due to the discitis and psoas abscess, she required a six-week antibiotic course and remained at a high risk of *C. diff* recurrence.

By early November 2024, the patient reported new episodes of loose foul-smelling stool. A repeat stool culture was taken on 5 November 2024 which was positive for recurrent *C. diff* infection, with both PCR- and toxin EIA-positive results—notably, CE 015 strain.

As per UK national guidelines, a second course of vancomycin was commenced [12]. After 48 h of vancomycin, there was a lack of symptom response; therefore, it was switched to fidaxomicin. This prompted a discussion with the microbiology team, who advised that this change was premature, given that vancomycin may take a week to show full effect. Vancomycin was resumed and the patient then completed a ten-day course.

Due to the prolonged hospital stay and multiple infections, the patient became significantly deconditioned. Prior to admission, she was independent with a clinical frailty score (CFS) of 3; however, she became largely bedbound within a few months. She remained in hospital for inpatient rehabilitation. In December 2024, she developed pseudogout and reactive arthritis which was treated with colchicine. Shortly after starting colchicine, she developed loose stool, and another *C. diff* PCR and toxin EIA were positive, marking her third episode.

She received fidaxomicin for her third episode of *C. diff* which resolved her symptoms. Given the recurrent infections, the gastroenterology and infectious diseases teams were consulted and recommended FMT. She was discharged on 10 February 2025 and underwent capsule FMT on 12 March 2025. She has reported sustained improvement and no further recurrence since the transplant.

A summarised timeline of events can be seen in Table 1.

### 2.2. Patient 2

Patient 2 is an 83-year-old female who was admitted on 3 August 2023 with a five-day history of non-bloody diarrhoea and dysuria. Initial investigations showed a C-reactive protein (CRP) level of 300, a White Cell Count (WCC) of 28 and an acute kidney injury with an estimated glomerular filtration rate (eGFR) of 21. A CT of the abdomen showed left-sided colonic wall thickening, vascular calcifications, and ascites, raising suspicion of ischaemic colitis. Surgical review favoured infective colitis and a conservative approach with co-amoxiclav, metronidazole, and gentamicin, and supportive measures were adopted.

On 8 August 2023 she developed worsening abdominal distension. Stool testing was initially *C. diff* PCR-negative. Gastroenterology review on 10 August 2023 suggested likely ischaemic colitis, with diverticulitis as a differential, and antibiotics were discontinued unless deterioration occurred.

By 16 August 2023, her symptoms persisted; repeat testing on 16 August 2023 confirmed *C. diff* PCR- and toxin EIA-positive. She then commenced oral vancomycin 125 mg four times a day to complete a 10-day course. Repeat CT showed “extensive colitis with oedematous transverse colon and thumb printing, though no perforation,” prompting the addition of IV metronidazole 500 mg three times a day.

The gastroenterology team reviewed the patient again on 21 August 2023, and noted that she had persistently elevated WCC and CRP despite being on day four of vancomycin and metronidazole. Following microbiology input on 22 August 2023, vancomycin was increased to 500 mg four times a day. On 30 August 2023, microbiology advised continuing high-dose vancomycin and withholding repeat stool testing, given its limited impact on management. Persistent symptoms were thought unlikely to be solely due to *C. diff* and may need further investigations.

By early September, she developed hospital-acquired-pneumonia and fluid overload secondary to heart failure. During this time, she also completed the course of vancomycin for a total of 14 days. Her diarrhoea was managed symptomatically with creon and loperamide. Despite transient improvement, the diarrhoea worsened significantly again by mid-September. Another *C. diff* sample was sent; however, no conclusive results were obtained. As the most recent episode occurred within the past 28 days, and the test was expected to remain positive, given the high clinical suspicion of relapse, treatment with fidaxomicin was initiated despite the lack of results.

Repeat CT abdomen and pelvis (CTAP) showed radiological improvement, but flexible sigmoidoscopy revealed pseudomembranes. As symptoms improved, she was discharged to a rehabilitation facility. FMT was considered but deferred at this time to allow her to build up physiological reserve prior to administration.

#### Second Admission

In early October, her symptoms recurred in rehabilitation. Stool culture on 17 October 2023 was positive for *C. diff* PCR and EIA toxin, prompting readmission. Repeated imaging showed pancolitis. She was restarted on oral vancomycin and IV metronidazole, with microbiology recommending a vancomycin taper: four times a day for two weeks, then three times a day for two weeks, then alternate days for six weeks.

Despite treatment, by 24 October 2023, she reported seven bowel movements in one day. The gastroenterology team suggested a repeat flexible sigmoidoscopy and discussion with St Thomas’ Hospital infectious diseases department for consideration of FMT.

A planned sigmoidoscopy on 31/10 showed no active inflammation; biopsies revealed mild, patchy active non-pseudomembranous colitis. Her symptoms resolved by 3 November 2023, and she was discharged on 8 November 2023.

On discharge, the patient was referred for gastroenterology outpatient review and to Guy’s and St Thomas’ Hospital infectious diseases team. At outpatient follow-up on 17 November 2023, she was deemed clinically stable as repeat sigmoidoscopy showed resolving pseudomembranes. She was thereafter seen in Guy’s and St Thomas’ Hospital, and after extensive infectious disease work-up, she was deemed to be a suitable candidate for FMT. Donor screening followed the current Healthcare Infection Society (HIS) guidelines [13]. She underwent FMT on 10 January 2024 via five oral capsules, resulting in marked clinical improvement. At her final follow-up in March 2024, her bowel habits were stable, and she was experiencing daily formed stools, indicating effective resolution.

A summarised timeline of events can be seen in Table 2.

## 3. Discussion

### 3.1. Diagnostic Methodology

*C. diff* diagnosis can be a complicated process due to the fact that colonisation does not always indicate active infection. Diagnosis relies on a combination of clinical presentation, acute diarrhoea with characteristic foul odour, fever, abdominal pain, leucocytosis, and laboratory confirmation [14]. According to UK Standards for Microbiology Investigations (SMI), no single assay suffices for accurate CDI diagnosis; instead, a two-stage approach is mandated. This begins with a sensitive glutamate dehydrogenase (GDH) enzyme immunoassay (EIA) or nucleic acid amplification test (NAAT), followed by a specific toxin A/B EIA if the first test is positive. In cases where GDH or NAAT are positive, but the toxin assay is negative, a third NAAT is recommended to resolve discordant results [15]. In each episode of suspected *C. difficile* infection, the patients discussed in this case report were tested first with a NAAT (PCR) assay, followed by a toxin A/B EIA. This two-step process was performed in accordance with UK SMI guidelines. This highlights the importance of adhering to a structured diagnostic algorithm to ensure accurate identification of true infection and to avoid unnecessary treatment in cases of mere colonisation.

Ribotyping is required for surveillance and outbreak investigations; however, it plays no role in diagnosis. Ribotyping helps to trace strain types and identify hypervirulent strains such as ribotype 027 which are associated with higher transmissibility, increased severity of disease and greater recurrence rates [16]. Therefore, ribotyping is only used in cases where information influences infection control [17].

Toxin A and B immunoassay tests are used to detect *C. diff* toxins. They are quick to perform and relatively cheaper. A *C. diff* immunoassay should not be performed alone as this lacks clinical sensitivity but is specific for CDI. Other methods such as a cell cytotoxicity neutralisation assay and faecal calprotectin can also be used [16].

### 3.2. Considerations of Pulsed and Tapered Approach

In the case of Patient 2, the course of *C. diff* was more prolonged; therefore, some suggestions were made for tapering doses of antibiotics to prevent recurrence. After discussion with the local microbiology team, a tapering regimen of vancomycin was suggested [18].

This involved a regimen consisting of vancomycin administered four times daily for one to two weeks, followed by administration twice daily for one week, once daily for one week, and subsequently every two to three days for a duration of two to eight weeks [18]. The rationale behind this approach is to target residual *C. diff* spores as the pulsed dosing allows vancomycin to act on newly germinating spores [18].

### 3.3. Patient Considerations and Comparisons with Literature

These two cases were chosen for publication and comparison due to the similarities in initial management and progression to recurrent infections. With similar demographics they provide examples as to the importance of adherence to national guidelines and early consideration of FMT.

Both patients were elderly females > 65 years of age, with limited frailty markers prior to admission. Both acquired the infection > 48 h post-admission, and hence these were healthcare-associated infections [16]. They were both treated initially with oral vancomycin, escalated to fidaxomicin on recurrence, with the help of multidisciplinary team (MDT) discussions and planning, and ultimately underwent oral capsule faecal microbiota transplants following persistent or relapsing symptoms. In both cases, management involved early input from microbiology and gastroenterology teams, with eventual clinical resolution post-FMT.

Key differences between the two cases are that Patient 1 had a more complex medical course, with *E. coli* bacteraemia, discitis, and prolonged antibiotic use. She experienced three confirmed episodes of CDI and required multiple treatment adjustments. FMT was arranged after her third episode due to high recurrence risk. In contrast, for Patient 2, diagnostic endoscopy played a more prominent role in her management, with flexible sigmoidoscopy revealing pseudomembranous colitis even when stool tests were negative. Her imaging was suspicious of toxic megacolon and required IV metronidazole alongside vancomycin. Her FMT was considered much later on, after a relapse during rehab.

When compared with published recurrence statistics, both cases demonstrated outcomes consistent with high-risk populations. Recurrence after an initial CDI episode occurs in approximately 15–30% of cases, increasing to around 60% (58.4%) in patients aged over 65 or those requiring further antibiotics [19]. FMT has been shown to be equally effective in patients ≥ 80 years of age, as demonstrated in a 2022 retrospective cohort study [20]. Overall survival at 52 weeks after FMT was 78.9% as compared to 89.7% in patients aged 18–79, demonstrating its efficacy even in the older population [20].

Another retrospective single-centre study investigated patients ≥ 85 years who underwent FMT for recurrent CDI between 2014 and 2022 [21]. Amongst 43 patients who were included in the study, 77% achieved resolution rate at 8 weeks following the first transplant [21]. Of those who did not initially respond, half achieved resolution after a repeated procedure, making the overall efficacy 88% [21]. This study also assessed frailty using the clinical frailty score. Patients who responded successfully had a median CFS of 6, indicating moderate frailty, whereas those who did not respond had a median CFS of 8, indicating severe frailty [21]. These findings suggest that FMT not only treats recurrent CDI but also contributes to improved functional status and reduced frailty progression over the long term.

These two cases highlight the practical challenges in managing *C. diff* infections in older adults with complex comorbidities. Despite appropriate antibiotic therapy, both patients suffered from severe recurrent CDI that caused significant deterioration in their functional status. In each case, FMT was considered only after the third episode of infection. The recent literature confirms the safety and efficacy of FMT in the older population, and in light of updated national and international guidance, consideration of FMT after the first or second recurrence may be warranted. Integrating discussion surrounding FMT into care pathways for older and frail patients could reduce recurrence rates, antibiotic exposure, and functional deterioration.

### 3.4. Challenges and Risk Factors

One of the most significant challenges in managing Patient 1 was balancing the need for long-term broad-spectrum antibiotics and the development and recurrence of CDI. This patient required a six-to-eight-week course of antibiotics to successfully treat discitis and a congruent psoas abscess. Continuous use of antibiotics is a recognised risk factor in developing CDI, due to the disruption of the gut microbiome [22]. In most cases, discontinuation of the causative agent is usually the first step in management; however, this was not possible without jeopardising resolution of the discitis and abscess. CDI has a 15–30% recurrence rate after the first course of antibiotics therapy; however, with continued antibiotic use, this rate will inevitably increase [19].

Age over 65 is frequently reported as an important independent risk factor in developing recurrent CDI [19]. In Patient 1’s case, the combination of advanced age and the requirement for prolonged antibiotic therapy significantly increased her risk of recurrence—a risk that ultimately materialised during the course of her illness.

Another consideration in hospital-associated CDI is thorough environmental decontamination. In cases of patients with CDI, high levels of spore contamination of floors, bedrails, medical equipment, commodes, and other surfaces have been observed [23]. These spores are highly resistant to many conventional cleaning methods including alcohol-based sanitizers, detergents, and standard surface cleaners [23]. As a result, enhanced cleaning protocols are necessary to effectively reduce environmental contamination, transmission, and reinfection [23].

### 3.5. Faecal Microbiome Transplant Indications and Success Rates

The British Society of Gastroenterology (BSG) and HIS produced the first FMT guidelines in 2018 with a comprehensive update in 2024 [13]. The guidelines now explicitly recommend FMT be considered after a first recurrence of CDI and offered to patients experiencing two or more recurrences, reflecting emerging evidence and clinical experience [13]. Importantly, there are no restrictions placed on patients based on health status and age, with the only contraindication being an anaphylactic food allergy [13]. Donor screening is another important factor in ensuring the safety and efficacy of FMT. According to UK BSG and HIS guidelines, potential donors undergo comprehensive assessment including detailed health questionnaires, risk factor interviews, and extensive blood and stool testing [13].

FMT carries a success rate of more than 90% in cases of recurrent CDI and can be delivered through multiple routes including oral capsules, endoscopic methods (either upper gastrointestinal delivery through nasojejunal tubes or lower gastrointestinal delivery via colonoscopy), or rectal enemas [24,25]. The method of administration should take into account patient preference, tolerance of invasive procedures, access to equipment and procedures, and patient status [25]. Oral capsules are the least invasive option with high patient adherence and satisfaction, and were used in both patients in this paper. Endoscopic administration carries higher procedural risks but provides better visualisation of the digestive tract, leading to high success rates [24]. Enema administration is a less effective route and may require repeated treatments to achieve comparable results [25]. Regardless of administration methods, positive results have been seen with the administration of FMT, providing a majority of patients good symptomatic relief and decrease in recurrence rates [25].

### 3.6. Lessons for Practice

Both cases highlight the challenges of managing recurrent CDI in elderly adults, particularly where comorbidities and prolonged antibiotic use are present. They also demonstrate the importance of early multidisciplinary involvement between microbiology, gastroenterology, and infectious diseases specialists.

In both cases, FMT was performed only after multiple recurrences, by which point both patients experienced prolonged hospitalisation, functional decline, and considerable morbidity. Given emerging evidence and updated guidance recommending FMT after the first recurrence [13], earlier consideration could have potentially reduced symptom duration, hospitalisation, and recurrence.

Patient 2’s case also emphasises the diagnostic complexity of CDI, where endoscopic findings ultimately confirmed pseudomembranous colitis despite initial negative stool assays. This underlines the need for ongoing clinical judgement and the role of endoscopy in unclear cases.

Overall, these two cases support a more proactive approach in managing recurrent CDI in elderly adults. Early escalation to FMT, especially in high-risk or frail patients, may reduce recurrence and improve outcomes. Continued attention to antimicrobial stewardship and infection control remains essential in preventing new and recurrent infections.

## 4. Conclusions

*Clostridioides difficile* infection is a major concern, particularly in older patients as they are more likely to experience severe disease with higher chance of recurrence and poor outcome. The above cases highlight the difficulties in managing older patients with comorbidities. As the initial response to standard antibiotic therapy with vancomycin and fidaxomicin were ineffective, both patients experienced relapse and required multidisciplinary input. Ultimately, FMT was offered and achieved resolution in both patients. This is consistent with the evidence of the effectiveness of FMT in recurrent *C. diff* infection. These cases underscore the importance of early recognition, adherence to national guidelines, a multidisciplinary team (MDT) approach, and timely consideration of FMT.

## Figures and Tables

**Table 1:** Timeline of Patient 1’s clinical course, including key events, investigations, and management.

Date/Period	Key Clinical Events and Findings	Investigations and Results	Treatment and Management
15 August 2024	Admitted after fall with back pain and generalised aches.	Blood cultures: *E. coli* bacteraemia.	IV co-amoxiclav (seven days) for presumed urinary tract infection (UTI).
Late August 2024	Persistent *E. coli* bacteraemia; onset of diarrhoea.	Urine cultures negative; renal ultrasound normal; *C. diff* PCR/toxin-negative	Continued IV antibiotics; diarrhoea attributed to amoxicillin sensitivity.
Early September 2024	Worsening back pain and diarrhoea.	CT: toxic megacolon and bowel wall thickening MRI: T12/L1 discitis and left psoas abscess.	Conservative management advised. Began prolonged antibiotics (co-trimoxazole, piperacillin-tazobactam, amoxicillin).
18 September 2024	New onset severe diarrhoea.	Stool PCR positive for *C. diff*.	Oral vancomycin course for 10 days resulting in symptoms improved.
5 November 2024	Recurrent loose stool after ongoing antibiotics.	Stool PCR and toxin positive for *C. diff* (CE 015).	Vancomycin for 10 days (brief fidaxomicin trial, then reverted).
24 December 2024	Third diarrhoeal episode.	Stool PCR and toxin positive for *C. diff*.	Fidaxomicin for 10 days leading to full resolution.
January–February 2025	Rehab period; deconditioned but stable.	—	Supportive therapy; no new antibiotics.
10 February 2025	Discharged from hospital.	—	—
12 March 2025	Preventive management of recurrent *C. diff*.	—	FMT at St Thomas’ Hospital→no recurrence since.

**Table 2:** Timeline of Patient 2’s clinical course, including key events, investigations, and management.

Date/Period	Key Clinical Events and Findings	Investigations and Results	Treatment and Management
8 August 2023	Worsening abdominal distension; initial concern for *C. diff*.	Stool sent—*C. diff* PCR-negative.	Continued metronidazole; no new antibiotics started.
16 August 2023	Persistent diarrhoea, no improvement.	Repeat stool: *C. diff* toxin positive. CT: extensive colitis, with possible toxic megacolon.	Started oral vancomycin 125 mg four times a day for 10 days, with IV metronidazole 500 mg three times a day.
21–30 August 2023	Ongoing systemic inflammation.	WCC and CRP persistently elevated.	Continued vancomycin; microbiology advised against repeat stool testing and to continue high-dose regimen.
5 September 2023	Completed vancomycin course.	—	Symptomatic treatment with Creon and loperamide.
Mid–September 2023	Recurrence of diarrhoea; relapse suspected.	Stool cultures sent; prior *C. diff* positive within 28 days. Treated for a relapse of *C. diff* based on clinical suspicion.	Started fidaxomicin 200 mg twice a day for 10 days, following microbiology advice.
17 September 2023	—	*C. diff* PCR-negative.	Continued symptomatic care; monitoring only.
Late September 2023	Improving symptoms.	CTAP: improving colitis. Sigmoidoscopy: pseudomembranes present.	FMT considered but deferred due to recovery. Discharged to rehab.
17 October 2023	Recurrence of diarrhoea in rehabilitation.	*C. diff* PCR-positive leading to readmission (18/10).	Restarted oral vancomycin and IV metronidazole. Started vancomycin taper: four times a day for 2 weeks, then three times a day for 2 weeks, followed by alternate day dosing for 6 weeks.
24–31 October 2023	Persistent loose stools.	Sigmoidoscopy (31/10): no active inflammation; biopsies—mild, patchy active colitis.	Continued vancomycin taper; discussed FMT with infectious diseases (ID) team.
3–8 November 2023	Clinical resolution.	—	Discharged with outpatient gastro and ID follow-up.
17 November 2023	Outpatient review—stable, improving.	Repeat sigmoidoscopy: resolving pseudomembranes.	Referred for FMT at Guy’s and St Thomas’.
10 January 2024	Underwent faecal microbiota transplant (oral capsule form).	Donor screened per HIS/GIS guidelines.	Five oral capsules administered. Significant clinical improvement.
March 2024 (Final Follow-up)	Complete resolution of *C. diff*-related symptoms.	—	No further antibiotics required; sustained recovery.

## Data Availability

The original contributions presented in this study are included in the article. Further inquiries can be directed to the corresponding author.

## Abbreviations

The following abbreviations are used in this manuscript:

CDI	*Clostridioides difficile* Infection
*C. diff*	Clostridioides difficile
UTI	Urinary Tract Infection
FMT	Faecal Microbiome Transplant
*E. coli*	*Escherichia coli*
IV	Intravenous
PCR	Polymerase Chain Reaction
EIA	Enzyme Immunoassay
UK	United Kingdom
CT	Computed Tomography
MRI	Magnetic Resonance Imaging
CFS	Clinical Frailty Score
CRP	C-reactive Protein
WCC	White Cell Count
eGFR	Estimated Glomerular Filtration Rate
mg	Milligrams
CTAP	Computed Tomography Abdomen and Pelvis
HIS	Healthcare Infection Society
ID	Infectious Diseases
SMI	Standards for Microbiology Investigations
GDH	Glutamate Dehydrogenase
NAAT	Nucleic Acid Amplification Test
MDT	Multidisciplinary Team
BSG	British Society of Gastroenterology

## References

[1] Kuijper E.J., Reubsaet F.A.G., Barbut F. (2023). Clostridium, Clostridioides, and Other Clostridia. ClinMicroNow.

[2] Eeuwijk J., Ferreira G., Yarzabal J.P., Robert-Du M. (2024). A Systematic Literature Review on Risk Factors for and Timing of Clostridioides Difficile Infection in the United States. Infect. Dis. Ther..

[3] Wang D., Dong D., Wang C., Cui Y., Jiang C., Ni Q., Su T., Wang G., Mao E., Peng Y. (2020). Risk Factors and Intestinal Microbiota: Clostridioides difficile Infection in Patients Receiving Enteral Nutrition at Intensive Care Units. Crit. Care.

[4] Cho D.H., Kim S.H., Jeon C.H., Kim H.T., Park K.J., Kim J., Kwak J., Kwan B.S., Kong S., Lee J.W. (2024). Clinical Outcomes and Treatment Necessity in Patients with Toxin-Negative Clostridioides difficile Stool Samples. Ann. Clin. Microbiol. Antimicrob..

[5] Piccioni A., Rosa F., Manca F., Pignataro G., Zanza C., Savioli G., Covino M., Ojetti V., Gasbarrini A., Franceschi F. (2022). Gut Microbiota and Clostridium difficile: What We Know and the New Frontiers. Int. J. Mol. Sci..

[6] Smits W.K., Lyras D., Lacy D.B., Wilcox M.H., Kuijper E.J. (2016). Clostridium difficile Infection. Nat. Rev. Dis. Primers.

[7] Shin J.H., High K.P., Warren C.A. (2016). Older Is Not Wiser, Immunologically Speaking: Effect of Aging on Host Response to Clostridium difficile Infections. J. Gerontol. Ser. A Biol. Sci. Med. Sci..

[8] Czepiel J., Krutova M., Mizrahi A., Khanafer N., Enoch D.A., Patyi M., Deptula A., Agodi A., Nuvials X., Pituch H. (2021). Mortality following Clostridioides difficile Infection in Europe: A Retrospective Multicenter Case-Control Study. Antibiotics.

[9] Fekety R., McFarland L.V., Surawicz C.M., Greenberg R.N., Elmer G.W., Mulligan M.E. (1997). Recurrent Clostridium difficile Diarrhea: Characteristics of and Risk Factors for Patients Enrolled in a Prospective, Randomized, Double-Blinded Trial. Clin. Infect. Dis..

[10] Kamboj M., Khosa P., Kaltsas A., Babady N.E., Son C., Sepkowitz K.A. (2011). Relapse versus Reinfection: Surveillance of Clostridium difficile Infection. Clin. Infect. Dis..

[11] Kelly C.P. (2012). Can We Identify Patients at High Risk of Recurrent Clostridium difficile Infection?. Clin. Microbiol. Infect..

[12] National Institute for Health and Care Excellence (NICE) (2021). Clostridioides Difficile Infection: Antimicrobial Prescribing. https://www.nice.org.uk/guidance/ng199/chapter/recommendations#choice-of-antibiotic.

[13] Mullish B.H., Merrick B., Quraishi M.N., Bak A., Green C.A., Moore D.J., Porter R.J., Elumogo N.T., Segal J.P., Sharma N. (2024). The Use of Faecal Microbiota Transplant as Treatment for Recurrent or Refractory Clostridioides difficile Infection and Other Potential Indications: Second Edition of Joint British Society of Gastroenterology (BSG) and Healthcare Infection Society (HIS) Guidelines. Gut.

[14] Scottish Microbiology & Virology Network, Scottish C. difficile Reference Service, Health Protection Scotland (2016). Recommended Protocol for Testing for Clostridium difficile and Subsequent Culture.

[15] Lee H.S., Plechot K., Gohil S., Le J. (2021). Clostridium difficile: Diagnosis and the Consequence of Over Diagnosis. Infect. Dis. Ther..

[16] McDonald L.C., Gerding D.N., Johnson S., Bakken J.S., Carroll K.C., Coffin S.E., Dubberke E.R., Garey K.W., Gould C.V., Kelly C. (2018). Clinical Practice Guidelines for Clostridium difficile Infection in Adults and Children: 2017 Update by the Infectious Diseases Society of America (IDSA) and Society for Healthcare Epidemiology of America (SHEA). Clin. Infect. Dis..

[17] Seth-Smith H.M.B., Biggel M., Roloff T., Hinic V., Bodmer T., Risch M., Casanova C., Widmer A., Sommerstein R., Marschall J. (2021). Transition from PCR-Ribotyping to Whole Genome Sequencing-Based Typing of Clostridioides difficile. Front. Cell. Infect. Microbiol..

[18] McFarland L.V., Elmer G.W., Surawicz C.M. (2002). Breaking the Cycle: Treatment Strategies for 163 Cases of Recurrent Clostridium Difficile Disease. Am. J. Gastroenterol..

[19] Song J.H., Kim Y.S. (2018). Recurrent Clostridium difficile Infection: Risk Factors, Treatment, and Prevention. Gut Liver.

[20] Nivet C., Duhalde V., Beaurain M., Delobel P., Quelven I., Alric L. (2022). Fecal Microbiota Transplantation for Refractory Clostridioides Difficile Infection Is Effective and Well Tolerated Even in Very Old Subjects: A Real-Life Study. J. Nutr. Health Aging.

[21] Montalto M., Gallo A., Agnitelli M.C., Pellegrino A., Lipari A., Pero E., Covino M., Landi F., Gasbarrini A., Cammarota G. (2023). Fecal microbiota transplantation for recurrent Clostridioides difficile infection in frail and very old patients. J. Am. Geriatr. Soc..

[22] Mada P.K., Alam M.U. (2024). Clostridiodes Difficile Infection. https://www.ncbi.nlm.nih.gov/books/NBK431054/.

[23] Gouliouris T., Brown N.M., Aliyu S.H. (2011). Prevention and Treatment of Clostridium difficile Infection. Clin. Med..

[24] Goldenberg S.D., Batra R., Beales I., Digby-Bell J.L., Irving P.M., Kellingray L., Narbad A., Franslem-Elumogo N. (2018). Comparison of Different Strategies for Providing Fecal Microbiota Transplantation to Treat Patients with Recurrent Clostridium difficile Infection in Two English Hospitals: A Review. Infect. Dis. Ther..

[25] Gulati M., Singh S.K., Corrie L., Kaur I.P., Chandwani L. (2020). Delivery Routes for Faecal Microbiota Transplants: Available, Anticipated and Aspired. Pharmacol. Res..

